# Atrial Fibrillation Burden: Assessment, Clinical Significance, and Therapeutic Implications

**DOI:** 10.3390/life16081378

**Published:** 2026-08-21

**Authors:** Konstantinos Grigoriou, Vasileios Lamprou, Nikolaos Ktenopoulos, Panagiotis Theofilis, Panagiotis Iliakis, Panayotis K. Vlachakis, Anastasios Apostolos, Antonios P. Antoniadis, Nikolaos Fragakis, Paschalis Karakasis

**Affiliations:** 1 Department of Pharmacology, Medical School, University of Athens, 75 Mikras Asias Avenue, 11527 Goudi, Greece; dinosgrigoriou@gmail.com (K.G.);; 2 Manchester Heart Centre, Manchester Royal Infirmary, Manchester University NHS Foundation Trust, Manchester M13 9WL, UK; 3 First Cardiology Department, School of Medicine, Hippokration General Hospital, National and Kapodistrian University of Athens, 11527 Athens, Greece; nikosktenop@gmail.com (N.K.);; 4 Department of Medicine, Division of Cardiology, Angiology and Internal Emergency Medicine, Ruhr University Bochum, Knappschaft Kliniken University Hospital Bochum, 44892 Bochum, Germany; 5 Department of Cardiology, Guy’s and St Thomas’ NHS Foundation Trust, Harefield Hospital, London UB9 6JH, UK; 6 Second Department of Cardiology, Hippokration General Hospital, Aristotle University of Thessaloniki, 54642 Thessaloniki, Greece

**Keywords:** atrial fibrillation, AF burden, rhythm control, catheter ablation, antiarrhythmic drugs

## Abstract

Atrial fibrillation (AF) is traditionally classified using categorical clinical patterns and binary recurrence endpoints. However, these measures do not fully reflect arrhythmia frequency, duration, temporal distribution, or progression. AF burden, defined as the proportion of monitored time spent in AF, provides a more granular measure of disease activity and therapeutic response. Recent advances in cardiac implantable electronic devices, insertable cardiac monitors, wearable technologies, and digital platforms have significantly improved burden assessment, although considerable heterogeneity remains in monitoring methods, definitions, and clinically relevant thresholds. This review examines contemporary approaches to AF burden measurement and its associations with thromboembolic risk, heart failure, hospitalization, symptoms, quality of life, disease progression, and mortality. We particularly discuss the reduction of AF burden as a potential therapeutic target following antiarrhythmic drug therapy and catheter ablation, and the limitations of conventional recurrence definitions based on episodes lasting more than 30 s. This review also focuses on the implications for rhythm-control selection, post-ablation monitoring, anticoagulation, and clinical-trial design, while proposing priorities for standardized burden-based assessment.

## 1. Introduction

AF is the most common sustained cardiac arrhythmia among adults worldwide. It raises the risk of stroke, heart failure (HF), hospitalization, impaired quality of life (QoL), cognitive decline, and it is associated with increased mortality [1,2]. Despite significant advances in understanding the pathophysiology of AF and establishing rhythm-control therapies, evaluating and diagnosing AF remains largely based on clinical classifications and binary outcome measures that were developed decades ago [3,4]. Current guidelines categorize AF according to episode duration and persistence into paroxysmal, persistent, long-standing persistent, or permanent forms [5]. Although these categories are useful in the clinical setting, they only provide a broad description of arrhythmia behavior and fail to capture the dynamic and heterogeneous nature of AF over time [3,6].

Similarly, the effectiveness of rhythm-control interventions has usually been assessed using recurrence-based endpoints, most commonly the occurrence of any documented AF episode lasting more than 30 s following treatment [7]. While this definition has facilitated comparisons across clinical trials, it has important limitations. Patients with significant improvement in symptoms, cardiac function and QoL, who experience substantial reductions in arrhythmia frequency and duration may still be classified as treatment failures because of a single brief recurrence. Conversely, patients with recurrent episodes of longer duration may have a substantially greater burden of disease and share the same binary outcome. Such observations highlight the shortcomings of recurrence-based assessments and underscore the need for more quantitative measures of AF activity [3].

**Figure 1:**
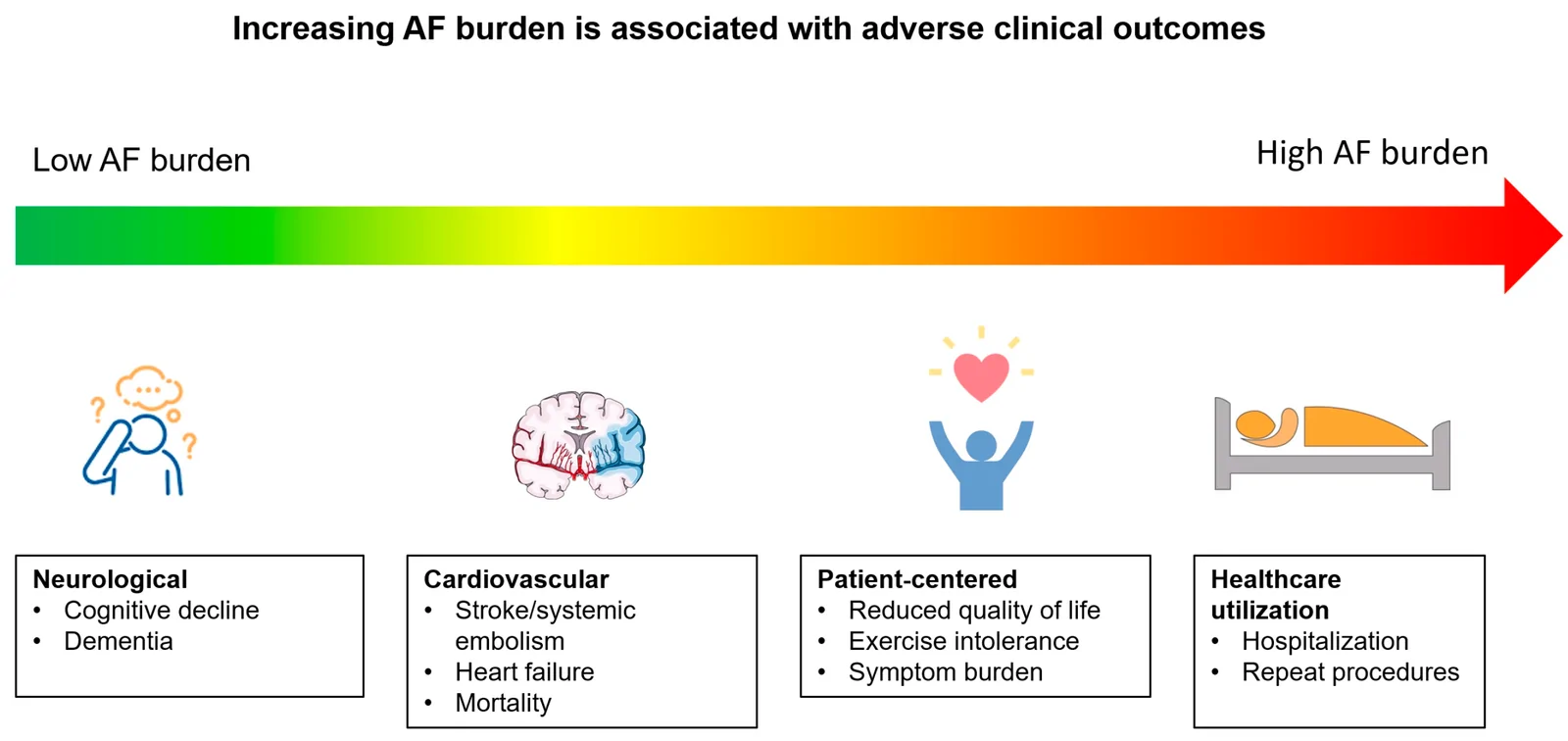
Increasing AF burden is associated with adverse clinical outcomes. The figure conceptually illustrates the progressive association between increasing AF burden and the risk of adverse clinical outcomes. AF burden seems to be an important marker of disease severity and prognostic risk. As AF burden rises, the incidence of outcomes such as stroke or systemic embolism, heart failure, hospitalization, and mortality generally increases. The figure is intended for illustrative purposes and does not represent validated quantitative AF burden thresholds or risk cut-offs. AF, atrial fibrillation.

AF burden, defined as the percentage of monitored time spent in AF, has emerged as a more refined measure of capturing the frequency, duration, temporal distribution, and progression of arrhythmia episodes [3]. Numerous studies have demonstrated that AF burden may represent a more adequate marker of the biological severity of the disease and of its clinical implications than the standard classification schemes. In fact, high AF burden is associated with poor outcomes such as stroke, HF, hospitalizations, and impaired QoL, whereas decrease of burden in response to AAD therapy or catheter ablation has been found to be more associated with clinically relevant benefits than absence of recurrence [8,9,10,11] (Figure 1).

Recent advancements of monitoring technologies have accelerated interest in AF burden assessment. Cardiac implantable electronic devices (CIEDs), insertable cardiac monitors, wearable sensors, and digital health platforms now permit precise characterization of arrhythmia patterns over prolonged periods. Nevertheless, substantial heterogeneity persists regarding monitoring strategies, burden definitions, reporting standards, and clinically meaningful thresholds, limiting the integration of burden-based metrics into routine practice and clinical research [3].

In this review, we examine contemporary approaches to AF burden measurement and summarize current evidence linking AF burden to clinically relevant outcomes. We discuss the role of burden reduction as a therapeutic target in rhythm-control strategies, evaluate the limitations of conventional recurrence-based endpoints, and explore the implications of burden-guided assessment for patient management, anticoagulation decisions, post-ablation monitoring, and future clinical-trial design.

## 2. Defining and Measuring AF Burden

### 2.1. Conceptual Definitions

AF burden as a term has referred to numerous definitions and there is not yet a single accepted definition. More recently, however, there has been consensus that AF burden is best described as the proportion of monitored time spent in AF during a specified observation period. This when expressed as a percentage, it reflects the cumulative duration of AF relative to total monitoring time [3]. For example, a patient who spends 24 h in AF during a 30-day monitoring period would have an AF burden of approximately 3.3%. Unlike traditional classifications, AF burden provides a continuous measure of arrhythmia activity and enables quantitative assessment of disease progression and therapeutic response [3,12]. To facilitate consistent clinical and research implementation, AF burden reporting should include the monitoring modality, total monitoring duration, method of AF detection, minimum analyzable recording time, and both absolute AF duration and relative burden (%). For appropriate interpretation, it is also important to report the longest AF episode duration and distinguish device-detected atrial high-rate episodes (AHRE) from clinically documented AF.

Despite its increasing clinical significance, AF burden is only one parameter of the arrhythmia behavior. Other parameters that are equally important and provide complementary information are AF episode duration, which describes the length of individual episodes, and AF frequency, which refers to the number of episodes occurring during a given observation period. The average or peak ventricular rate during AF episodes, and the frequency and duration of pauses occurring during AF or during transitions between sinus rhythm and AF characterize further the arrhythmic phenotype. Such parameters may vary significantly in patients with similar overall AF burden and may indicate distinct pathophysiological and clinical implications [3,12].

An additional classification of AF behavior is the temporal organization of AF episodes that may occur sporadically, cluster within relatively short time intervals, or become increasingly sustained as the disease progresses. This phenomenon is better described by the concept of AF density [13]. AF density quantifies the degree to which AF episodes are aggregated within the monitoring period. For example, high-density AF is characterized by clustering of episodes, whereas low-density AF reflects a rather uniform distribution. Although AF density remains primarily a research tool, it may provide important insights into disease dynamics that are not captured by burden alone.

Compare to burden alone, these complementary metrics provide a more comprehensive characterization of AF and may assist the clinician to interpret monitoring data, better assess disease progression, and evaluate the rhythm-control therapies as monitoring technologies continue to evolve.

### 2.2. Monitoring Technologies

#### 2.2.1. Intermittent Monitoring

Intermittent rhythm-monitoring modalities such as symptom-triggered electrocardiogram (ECG), ambulatory Holter monitoring, external event recorders, and adhesive patch monitors remain important tools for AF detection and follow-up in routine clinical practice. Their widespread availability, non-invasive nature, and relatively low cost have made them indispensable for diagnosing AF and assessing treatment efficacy [14].

However, since intermittent monitoring capture only a fraction of the total observational period, it is an inherently limited method for quantifying AF burden [15]. Therefore, it is strongly influenced by monitoring duration and timing. Furthermore, short or asymptomatic episodes may remain undetected, whereas recordings obtained during periods of increased arrhythmia activity may overestimate long-term disease severity [3].

The effectiveness of intermittent monitoring is enhanced by recording intervals. Prolonged Holter monitoring, adhesively applied monitor patches, and external event recorders enable the recording of the rhythm for several days or even weeks. This enhances true AF burden estimation [16]. Nevertheless, none of these modalities provides uninterrupted long-term rhythm assessment.

#### 2.2.2. Continuous Monitoring

Continuous rhythm monitoring provides near-complete temporal characterization of atrial arrhythmia episodes and allows accurate assessment of their frequency, duration, temporal distribution, and progression over time. It also minimizes sampling error and captures asymptomatic episodes that are a large proportion of AF recurrences. These make this method the current reference standard for AF burden assessment. It is important to note that AF burden should always be reported together with the duration of monitoring, as the monitored time constitutes the denominator for burden calculation. Current consensus also recommends reporting the duration of the longest uninterrupted AF episode, because this complementary metric provides additional information about arrhythmia behavior that may not be reflected by AF burden alone [3].

##### Cardiac Implantable Electronic Devices

CIEDs include permanent pacemakers, implantable cardioverter-defibrillators (ICDs), and cardiac resynchronization therapy (CRT) devices, and provide continuous atrial rhythm surveillance in patients with implanted atrial leads [17,18]. Modern devices can detect atrial tachyarrhythmias with high sensitivity and specificity because they use sophisticated algorithms based on atrial rate, rhythm regularity, electrogram morphology, and mode-switch episodes [19,20,21]. Additionally, remote monitoring of patients with CIEDs has enhanced the resolution of diagnostic information [22].

Despite their high diagnostic accuracy, CIEDs have some limitations in AF detection. Inaccurate AF detection by CIEDs is more common with episodes lasting < 2 min. In contrast, episodes that persist > 6 min are more likely to represent true AF. In addition, sensing interruptions may lead to document a single prolonged AF episode into multiple shorter episodes. This may affect episode counts [3]. Failure to capture brief AF episodes is unlikely to meaningfully influence the calculated AF burden, unless such episodes occur with high frequency [23].

##### Insertable Cardiac Monitors

Insertable cardiac monitors (ICMs) extend continuous rhythm surveillance to patients who do not have conventional pacing indications. Compared with external monitoring modalities, ICMs provide prolonged monitoring, improve the detection of asymptomatic AF episodes, reduce reliance on patient participation, and, as a result, enable more accurate quantification of AF burden. Furthermore, the integration of artificial intelligence (AI)-based algorithms has substantially reduced false-positive AF detections while maintaining diagnostic sensitivity [24]. ICMs may support clinical decision-making regarding rhythm-control strategies and anticoagulation therapy in both symptomatic and asymptomatic patients, including those with cryptogenic stroke [25,26,27].

However, ICMs have some limitations. Their accuracy may be limited by signal quality obtained from subcutaneous ECG recordings. Artifacts, inadequate R-wave sensing, and oversensing or undersensing events can lead to inappropriate detections, which include false pauses, high-rate episodes, or erroneous AF classification due to altered RR variability [28,29]. Optimizing implantation technique and device positioning to achieve stable R-wave sensing, together with improved sensing algorithms, remains important to minimize these limitations and enhance the reliability of long-term AF burden assessment [30].

Overall, while continuous monitoring has inherent limitations, it remains the most comprehensive approach for longitudinal assessment of AF burden. The ability of studying temporal trends and obtaining objective measurements offers us valuable insights about the course of disease and effectiveness of therapy. However, the results should be always interpreted in the context of the individual characteristics of each patient.

#### 2.2.3. Wearables and Digital Health

The widespread availability of consumer-grade digital health devices has given us the opportunity to expand AF detection beyond conventional medical monitoring. Wearable technologies, like smartwatches and handheld devices, enable on-demand single-lead ECG recordings or passive surveillance of pulse irregularity using photoplethysmography (PPG). These technologies provide a scalable approach for rhythm assessment and longitudinal monitoring, allowing detection of intermittent or asymptomatic AF episodes in large populations [31,32].

PPG-based wearables identify irregular pulse patterns mainly because they can detect peripheral blood volume changes and are primarily used for AF screening [33,34,35]. However, because PPG does not directly assess atrial electrical activity, diagnostic accuracy varies across devices and may be affected by motion artefacts, premature atrial contractions, skin pigmentation, and intermittent recordings, resulting in both false-positive and false-negative detections and potential underestimation of AF burden [36,37,38,39].

Consumer ECG devices, including smartwatch-integrated and handheld single-lead systems, provide direct electrical rhythm recordings that can be reviewed by a healthcare professional, making them particularly useful in patients at higher risk of AF or with cardiovascular disease. However, because ECG recordings require user activation, these devices are less suitable for continuous rhythm monitoring than PPG-based wearables [40].

Most consumer wearables do not provide continuous rhythm monitoring and are therefore not suitable for precise AF burden quantification, unlike CIEDs and ICMs. Nevertheless, validated ECG- or combined PPG/ECG-based wearables may enable longitudinal assessment of intra-individual AF burden trends and support remote follow-up and patient-centered rhythm management [3].

It is important to mention that AF burden estimates are influenced by monitoring duration, device technology, detection algorithms, thresholds, and reporting methods [3]. Therefore, measurements obtained from continuous monitoring devices cannot be directly compared with those derived from intermittent ECG or wearable-based approaches, because these modalities differ fundamentally in sampling density and the proportion of the total observation period included in the burden calculation. Consequently, intermittent monitoring provides sampling-derived burden estimates that are more susceptible to classification bias than continuously acquired measurements. Standardized definitions and reporting frameworks are needed to ensure meaningful interpretation across different monitoring strategies.

## 3. AF Burden and Disease Biology

### AF Burden as a Marker of Atrial Cardiomyopathy

AF burden has been shown to represent a quantifiable proxy of the atrial disease process, rather than being merely a reflection of arrhythmia incidence [3]. Increasing burden reflects progressive alterations in atrial structure, electrophysiology, and molecular biology that collectively define atrial cardiomyopathy [12,41]. An increase in AF burden promotes further remodeling, creating a self-perpetuating cycle in which burden both mirrors and accelerates disease progression [2,4,42]. This bidirectional relationship provides the biological rationale for considering AF burden as a therapeutic target rather than solely a clinical endpoint.

Structural remodeling develops through chronic exposure to cardiovascular risk factors, including hypertension, obesity, diabetes mellitus (DM), HF, and aging, resulting in atrial enlargement, myocyte hypertrophy, extracellular matrix expansion, and progressive interstitial fibrosis [41,43]. These alterations affect the atrial myocardial architecture, slow conduction velocity, and promote conduction heterogeneity that facilitates re-entry and promotes AF maintenance [44]. With increasing fibrosis, episodes of AF become progressively more prolonged and frequent and increasingly resistant to spontaneous termination, thus progressively increasing the AF burden [45,46,47]. There is significant advancement in the cardiac imaging field regarding the understanding of the fibrotic substrate of the atrium. With the help of cardiac magnetic resonance (CMR) imaging, atrial fibrosis is assessed by late gadolinium enhancement (LGE) and T1 mapping, whereas electroanatomical voltage mapping detects functional alterations in the heart [41]. The low voltage areas (LVAs), an electrophysiological marker for atrial fibrosis, have a strong association with recurrent AF post-catheter ablation [48].

Inflammation and oxidative stress are additional biological mechanisms that are linked with an increasing AF burden and progressive atrial remodeling. Elevated circulating concentrations of inflammatory mediators, including C-reactive protein (CRP), interleukin (IL)-6, IL-17, human transforming growth factor beta 1 (TGF-β1), tumor necrosis factor-α (TNF-α), and activation of the NOD-, LRR-, and pyrin domain-containing protein 3 (NLRP3) inflammasome pathway, have been associated with both incident AF and greater arrhythmia burden [49,50,51,52,53]. Inflammatory signaling promotes fibroblast activation, extracellular matrix remodeling, and abnormalities of intracellular calcium (Ca^2+^) handling, thereby creating a substrate favorable for AF initiation and maintenance [41]. Oxidative stress similarly promotes structural and electrical atrial remodeling, with increased reactive oxygen species (ROS) production, particularly through NADPH oxidase (NOX) activation and mitochondrial dysfunction, driving atrial cardiomyopathy, while downstream lipid peroxidation products such as isolevuglandins (IsoLGs) further exacerbate molecular injury and amyloid formation, increasing AF susceptibility [41,54]. AF itself causes atrial stretch, oxidative stress, mitochondrial dysfunction, and activation of pro-inflammatory pathways, amplifying the inflammatory response [41,55]. AF burden is not only a consequence of inflammation and oxidative stress but may actively sustain this milieu that drives further substrate progression.

Electrical remodeling develops rapidly following the onset of AF and contributes substantially to increasing the arrhythmia burden [56]. Multiple mechanisms contribute to its development, including altered gene expression, autonomic nervous system (ANS) dysfunction, impaired ion channel function, inflammatory processes, and oxidative stress. Its primary electrophysiological features include a reduction in both the atrial effective refractory period (AERP) and action potential duration (APD), decreased atrial conduction velocity, and increased heterogeneity of refractoriness. These alterations promote the formation of localized electrical activity and multiple reentrant circuits within the atria, thereby creating a substrate that facilitates both the initiation and maintenance of AF [57]. Importantly, while early electrical remodeling may be partially reversible following restoration of sinus rhythm, chronic exposure to high AF burden promotes irreversible structural changes that reduce the likelihood of durable rhythm control [41,58,59].

The concept that AF burden is an integrative parameter characterizing atrial cardiomyopathy as an outcome of structural, fibrotic, inflammatory, and electrical remodeling is increasingly recognized. At the same time, a greater AF burden plays a role in the deterioration of the atrial substrate through the remodeling process [60]. The above-mentioned bidirectional relationship provides a rationale to target the AF burden reduction as a therapeutic goal, especially in the early stages of the disease when remodeling of the atrium is still reversible. Thus, AF burden should be considered not only as a parameter characterizing the frequency of arrhythmias but also as a biomarker of atrial condition.

## 4. Clinical Significance of AF Burden

### 4.1. AF Burden and Stroke Risk: From Binary Diagnosis to Quantitative Risk Assessment

The link between AF burden and thromboembolic risk has emerged as one of the most clinically relevant aspects of contemporary AF management. It is logical to hypothesize that stroke risk in patients depends on AF burden in addition to established clinical risk factors. Whether quantitative measures of AF burden can improve thromboembolic risk stratification and guide personalized anticoagulation decisions beyond established clinical risk scores is under investigation.

#### 4.1.1. Evidence from Device-Based Studies

The ASSERT (ASymptomatic AF and Stroke Evaluation in Pacemaker Patients and the AF Reduction Atrial Pacing) trial provided the first major evidence linking AF burden to stroke risk. This study showed that in patients without previously diagnosed AF and with implanted pacemakers or defibrillators, AHRE that last at least 6 min were associated with approximately a 2.5-fold increase in the risk of ischemic stroke or systemic embolism. Subsequent analyses demonstrated that stroke risk increased substantially among patients who experienced episodes exceeding 24 h. Shorter episodes were associated with considerably lower absolute event rates [61]. These findings suggested that arrhythmia duration is clinically relevant and challenged the notion that all AF episodes carry similar prognostic significance.

The TRENDS study, which prospectively evaluated AF burden in patients with CIEDs and established stroke risk factors showed that patients with a daily AF burden exceeding approximately 5.5 h exhibited nearly double the annual thromboembolic risk compared with patients without AF, whereas those with lower burdens had intermediate risk. Although the increase did not reach statistical significance because of limited event numbers, the study introduced the concept that thromboembolic risk increases progressively with cumulative AF exposure rather than after a predefined episode duration [62].

The association between AF burden and thromboembolic risk was further strengthened by a pooled patient-level analysis of 10,016 patients from five prospective studies, that included the TRENDS study, involving individuals with CIEDs (SOS AF study). AF burden independently predicted ischemic stroke after adjustment for CHADS_2_ (congestive HF, hypertension, age ≥ 75 years, DM, stroke/transient ischemic attack/thromboembolism) score and baseline anticoagulation use during a median follow-up of 24 months. Among several prespecified burden thresholds (5 min, 1, 6, 12, and 23 h), a maximum daily AF burden of ≥1 h was associated with the greatest increase in stroke risk. It is important to note that the analysis indicates that AF burden is a valuable indicator of prognosis beyond conventional clinical risk scores, which supports the concept that quantitative assessment of AF burden may improve thromboembolic risk stratification in patients with AF. Nevertheless, the lack of an abrupt change in the relationship between burden levels and stroke rate suggests that risk may increase gradually and not at some arbitrary threshold point [63].

The KP-RHYTHM study extended these observations using prolonged ambulatory monitoring in patients with paroxysmal AF. The study demonstrated that the percentage of monitored time spent in AF was independently associated with thromboembolic events after adjustment for CHA_2_DS_2_-VASc score (congestive HF, hypertension, age ≥ 75 years, DM, stroke/transient ischemic attack/thromboembolism, vascular disease, age 65–74 years, sex category) and other clinical variables. Patients in the highest tertile of AF burden experienced significantly greater stroke risk than those with lower burdens. This finding supports AF burden as a continuous marker of disease severity [8].

Collectively, these observational studies suggest that increasing AF burden reflects progressively greater thromboembolic risk and also demonstrate substantial overlap between burden categories, indicating that AF burden alone is insufficient to determine individual stroke risk.

#### 4.1.2. Randomized Evidence from Screening and Anticoagulation Trials

Recent randomized clinical trials (RCTs) have further refined our understanding of the association of device-detected AF burden and stroke prevention.

The LOOP trial studies continuous monitoring with ILRs in elderly individuals with an increased risk of stroke. When patients received continuous monitoring, their detection of AF and initiation of anticoagulation was significantly increased. However, this strategy did not significantly reduce the incidence of stroke or systemic arterial embolism compared with standard care. These findings indicate that identifying short-duration subclinical AF alone may be insufficient to improve clinical outcomes and emphasize that not all detected AF episodes warrant identical therapeutic responses [64].

More recently, two complementary RCTs have addressed anticoagulation in patients with device-detected subclinical AF.

The NOAH-AFNET 6 trial assessed whether anticoagulation with edoxaban prevented cardiovascular events in patients with AHREs but without electrocardiographically documented AF. The trial was terminated early as anticoagulation failed to reduce the composite endpoint of stroke, systemic embolism, or cardiovascular death while significantly increasing major bleeding. These findings argue against routine anticoagulation solely on the basis of short-duration device-detected AHRE [65].

In the ARTESiA trial apixaban was compared with aspirin in patients who had device-detected subclinical AF lasting between 6 min and 24 h and also had elevated stroke risk. Apixaban lowered the ischemic stroke and systemic embolism rates but increased major bleeding. Although the absolute reduction in stroke was modest, ARTESiA showed that selected high-risk patients with subclinical AF derive measurable benefit from anticoagulation. This finding underscores the importance of integrating AF burden with overall clinical risk [66]. The individual results of NOAH-AFNET 6 and ARTESiA may appear contradictory. However, a subsequent meta-analysis that included 6548 patients demonstrated consistent treatment effects. Oral anticoagulation with edoxaban or apixaban reduced ischemic stroke by 32% and increased major bleeding by 62%, without a significant effect on cardiovascular or all-cause mortality. These findings suggest that anticoagulation can reduce thromboembolic events in appropriately selected patients with device-detected AF and reinforce the importance of individualized assessment of both stroke and bleeding risk [67].

Taken together, NOAH-AFNET 6 and ARTESiA suggest that AF burden may contribute to our decisions regarding anticoagulation in selected patients, but clinical thromboembolic risk remains the primary determinant. The variation in findings likely reflect differences in patient populations, endpoint definitions, anticoagulant selection, and underlying stroke risk rather than fundamentally contradictory biological mechanisms.

#### 4.1.3. Is There a Threshold of AF Burden?

A significant unresolved question is whether a clinically meaningful AF burden threshold exists above which stroke risk rises enough to justify anticoagulation.

The previously described studies have suggested possible AF burden thresholds ranging from 6 min to several hours of daily AF or cumulative episodes exceeding 24 h. These cutoffs largely reflect study-specific definitions and not discrete biological transitions in thromboembolic risk. ASSERT identified an important increase in stroke risk among patients with episodes lasting > 24 h. TRENDS suggested higher thromboembolic risk with a daily AF burden exceeding approximately 5.5 h. Importantly, these seemingly separate findings were reconciled by a further systematic review and dose–response meta-analysis of 16 studies, which included ASSERT, TRENDS, and the SOS AF project and had more than 53,000 patients. This study showed that AF episodes which last > 5 min were associated with a significantly increased risk of stroke and, more importantly, identified a linear dose–response relationship. The stroke risk increased by approximately 2% for every additional hour of AF burden. These data suggest that thromboembolic risk increases progressively with greater AF burden and support the concept that AF burden is a continuous rather than a dichotomous risk factor [9].

#### 4.1.4. Interaction Between AF Burden and CHA_2_DS_2_-VASc Score

Increasing evidence suggests that AF burden and CHA_2_DS_2_-VASc score provide complementary rather than competing prognostic information. It seems that clinical risk factors remain the dominant determinants of thromboembolic risk, while AF burden appears capable of changing that baseline risk [68]. Thus, patients with high CHA_2_DS_2_-VASc scores have high stroke risk even with relatively limited AF burden, whereas younger individuals with low clinical risk may remain at low absolute risk despite prolonged AF episodes. Quantitative assessment of AF burden may refine estimates of thromboembolic risk and potentially influence clinical decisions regarding anticoagulation especially among intermediate-risk patients [3].

Current guidelines continue to prioritize clinical thromboembolic risk scores for anticoagulation decisions. AF burden is acknowledged as an additional factor that requires individualized interpretation, particularly in patients with subclinical AF detected through long-term monitoring. However, whether it should independently guide anticoagulation decisions remains an area of ongoing investigation.

### 4.2. AF and Cognitive Impairment

Accumulating evidence indicates that AF independently increases the risk of cognitive decline, beyond its association with stroke [69]. Several pathophysiological mechanisms have been proposed to explain this association, including cerebral hypoperfusion, silent microembolic events, impaired cardiac output, abnormal endothelial shear stress, neuroinflammation, and the potential adverse effects of AF-related medications [70,71].

The role of AF burden in the development and progression of AF-related cognitive decline remains uncertain and the available evidence is largely derived from small studies. In a prospective study of 30 patients with symptomatic paroxysmal AF undergoing catheter ablation and continuous rhythm monitoring with an ILR, an AF burden ≥ 0.5% was not associated with worse overall cognitive performance over six months compared with an AF burden < 0.5%. Although patients with a lower AF burden demonstrated a trend toward greater improvement in verbal learning and visual memory, these differences did not reach statistical significance, and AF burden had no significant overall impact on cognitive function during follow-up [72]. A larger prospective cohort study of 253 patients with non-valvular AF using 14-day patch-based ECG monitoring demonstrated a significant inverse association between AF burden and cognitive function assessed by the Montreal Cognitive Assessment (MoCA). Higher AF burden was independently associated with lower MoCA scores after adjustment for demographic characteristics, comorbidities, and echocardiographic parameters, suggesting that greater AF burden may contribute to cognitive impairment in patients with AF. However, further studies are needed to determine whether reducing AF burden can prevent or slow cognitive decline [73].

Furthermore, in the AGES-Reykjavik Study of 2291 elderly individuals, higher AF burden, reflected by persistent rather than paroxysmal AF, was associated with reduced cerebral blood flow and lower brain perfusion compared with individuals without AF. These findings suggest that greater AF burden may contribute to impaired cerebral perfusion, potentially linking AF progression with cognitive decline [74].

### 4.3. AF Burden and Heart Failure

HF and AF share a complex bidirectional association in which each condition can lead to the development and progression of the other. AF may cause or exacerbate HF through loss of atrial mechanical function, atrioventricular dyssynchronization, rapid ventricular rates, increased intracellular production of ROS, impaired left ventricular (LV) Ca^2+^ handling, and electrical remodeling. Neurohormonal activation and elevated ventricular filling pressures in HF create a highly arrhythmogenic background that facilitates AF initiation and maintenance [75,76]. In patients with HF, persistent and permanent AF are observed more often than paroxysmal AF, indicating a close relationship between HF and advanced AF phenotypes [75]. An observational study showed that long-standing persistent and permanent AF represent the predominant AF phenotypes, accounting for approximately 40–50% of cases, whereas paroxysmal and persistent AF each comprise around 20–30% of presentations [77].

Arrhythmia-induced cardiomyopathy (AIC) may lead to progressive deterioration of LV systolic function and has been described in as many as 50% of patients with AF. AIC is one of the clearest manifestations of prolonged AF exposure and it is thought to arise through multiple interrelated mechanisms, including systemic inflammation, oxidative and metabolic stress, impaired cardiomyocyte Ca^2+^ handling, reduction of coronary blood flow, and adverse structural remodeling [78,79]. These complex mechanisms indicate that AIC is not simply a consequence of tachycardia and that timely initiation and maintenance of effective rhythm-control strategies may play an important role in both preventing and reversing AIC [80,81]. Reduction of AF burden may reverse several key pathophysiological factors underlying AIC. For example, increasing AF burden has been associated with progressive impairment of coronary blood flow, whereas restoration of sinus rhythm can improve coronary perfusion [82,83]. However, the AF burden threshold required to induce AIC remains undefined, and the relationship between cumulative arrhythmic exposure and ventricular dysfunction has yet to be fully characterized.

While the interaction between AF and HF has traditionally been examined in patients with HF with reduced ejection fraction (HFrEF), increasing attention has focused on HF with preserved ejection fraction (HFpEF), where AF is a particularly prevalent risk factor [77,84]. AF is reported in approximately one-half of patients with HFpEF, although its prevalence may be underestimated due to undetected asymptomatic episodes without prolonged rhythm monitoring [85]. AF and HFpEF share common comorbidities and pathophysiological mechanisms, including advanced age, hypertension, obesity, diastolic dysfunction, coronary microvascular dysfunction, myocardial fibrosis, oxidative stress, and inflammation [84,86,87]. AF pattern appears to correlate with HFpEF prevalence, although direct evidence linking quantified AF burden with HFpEF remains limited. Permanent AF has been linked with a higher prevalence of HFpEF compared with paroxysmal AF, suggesting that cumulative arrhythmic exposure may contribute to HF development and progression [88,89]. A study in patients with HFpEF showed that increasing AF burden was associated with progressive deterioration of left atrial (LA) mechanics, including reduced LA compliance and reservoir function, as well as worsening hemodynamics and survival [90]. Interestingly, an AF burden threshold greater than 10.87% was associated with an increased risk of major adverse cardiovascular events (MACE) and worsening HF hospitalizations in patients with preserved or mildly reduced ejection fraction. However, it did not significantly alter the hazard ratio in patients with baseline reduced ejection fraction, likely because myocardial damage was already advanced in this group [91].

An increasing burden of AF is associated with adverse clinical outcomes in patients with HF. Studies employing CIEDs showed more direct evidence that higher AF burden is independently associated with an increased risk of HF hospitalization and mortality among patients with established HF [91,92]. In patients with sinus node dysfunction who had dual-chamber pacemakers or defibrillators and no history of AF, a daily AHRE burden of ≥6 min was associated with more than a fourfold increase in the risk of MACEs. This was mainly driven by cardiovascular death, worsening HF hospitalizations, and progression to sustained AF. In contrast, this association was not observed in patients with pre-existing AF [93].

Interestingly this relationship appears to also extend to individuals without established HF. As shown in an analysis of a large Medicare cohort the increasing AF burden was independently associated with a higher incidence of new-onset HF, supporting the concept that prolonged arrhythmic exposure may contribute directly to ventricular dysfunction even before overt HF develops [10].

In a sub-analysis of the ASSERT study, progression of subclinical AF to episodes lasting > 24 h were associated with an approximately five-fold increased risk of HF hospitalization, suggesting that increasing AF burden may represent an important marker of HF risk [94].

Despite increasing evidence, several important knowledge gaps remain as no universally accepted AF burden threshold has been identified that predicts HF onset, ventricular dysfunction, hospitalization, or mortality. Furthermore, substantial heterogeneity in monitoring strategies, burden definitions, and reporting methods limits comparisons across studies. Future investigations incorporating continuous rhythm monitoring and standardized AF burden metrics are needed to establish clinically actionable burden thresholds that can guide rhythm-control strategies and identify the patients that are most likely to benefit from early intervention.

### 4.4. AF Burden, Healthcare Utilization, and Quality of Life

The association of AF with impaired QoL is well established as it is affected both directly and through its interaction with comorbidities and other contributing factors. Studies are showing that approximately one in five patients experience a clinically meaningful decline in health status and QoL even over relatively short follow-up periods [95,96,97]. Improving QoL has therefore become a principal goal of rhythm-control strategies, although historically most clinical trials evaluating rhythm-control therapies have relied on AF recurrence as the primary efficacy endpoint, despite growing evidence that this measure correlates poorly with patient-reported outcomes [3].

In a subanalysis of the CAPTAF trial that included 150 patients with continuous rhythm monitoring using implantable cardiac monitors, AF burden was the most linked characteristic to poorer health-related QoL. After adjusting for clinical factors, AF burden remained independently connected to lower Short Form (SF)-36 Vitality scores, while AF episode duration and frequency did not show this connection. Specifically, a 10% increase in AF burden corresponded to a 1.34-point decrease in the Vitality score. This indicates that AF burden matters more for patient well-being than the duration or frequency of AF episodes. However, these results have not been consistently confirmed. In the MANTRA-PAF trial, which compared radiofrequency catheter ablation (RFA) to AAD therapy as the first treatment for paroxysmal AF over five years, patients undergoing RFA experienced significantly greater freedom from AF and a lower overall AF burden than those receiving AAD. Yet, even though QoL improved significantly from baseline in both groups, no major differences appeared between them. This suggests that reducing AF burden does not always lead to better patient-reported QoL [98,99].

In a prospective study of 346 patients undergoing catheter ablation with continuous rhythm monitoring, AF burden was more strongly associated with clinically meaningful outcomes than the conventional definition of recurrence (≥30 s). Patients with an AF burden > 0.1% had significantly higher healthcare utilization, including increased risks of emergency department visits, hospitalization, cardioversion, and repeat ablation, whereas those with an AF burden ≤ 0.1% had healthcare utilization comparable to patients without recurrence. These findings are suggestive that AF burden is a more clinically relevant marker compared to brief episodes of recurrent AF and may better identify patients at risk for increased healthcare resource utilization after catheter ablation [100].

Although the available evidence suggests that AF burden is a more clinically meaningful metric than conventional AF recurrence for predicting healthcare utilization and, in some populations, patient-reported QoL, the association between AF burden and QoL is not uniform across studies, indicating that symptom perception, comorbidities, and treatment strategy significantly contribute to patient well-being.

### 4.5. AF Burden, Cardiovascular and All-Cause Mortality

AF is linked with an increased risk of mortality, primarily due to its association with stroke and HF [101]. This risk appears to increase progressively with greater AF burden. In a large cohort of 21,391 patients with CIEDs, device-detected AF was independently associated with increased mortality. Compared with patients without AF, those with AF had a higher adjusted risk of all-cause mortality. Among patients with AF, greater arrhythmia persistence was associated with progressively higher mortality risk, with persistent and permanent AF demonstrating higher mortality compared with paroxysmal AF [102].

AF burden-mortality relationships have previously been highlighted also in critically ill patient populations. In a recent retrospective study assessing in-hospital AF burden in ICU survivors, patients with higher levels of AF burden (≥62.43%) were found to be at significantly greater 1-year all-cause mortality as compared to patients with lower burden of AF [13]. A linear relationship was observed for the impact of an increase in AF burden on mortality risk, however no significant association was seen with respect to 30-day mortality, suggesting further clarification is needed from long term follow up [103].

Supporting the importance of AF progression, a systematic review of 12 studies including 99,996 patients demonstrated that non-paroxysmal AF was associated with a higher risk of thromboembolism and all-cause mortality compared with paroxysmal AF [104].

Beyond AF pattern, symptom status has been evaluated as a potential surrogate of AF burden and clinical risk. However, symptoms do not reliably reflect the underlying arrhythmic burden, as many patients with significant AF burden remain asymptomatic. A recent meta-analysis of more than 217,000 patients found no significant differences in mortality, stroke, thromboembolism, myocardial infarction, or hospitalization between symptomatic and asymptomatic AF. These findings support the need for objective AF burden assessment rather than reliance on symptom status alone for risk stratification [105].

Overall, most evidence linking AF burden with adverse clinical outcomes derives from observational studies and populations with cardiovascular disease who undergo continuous monitoring. These cohorts may differ from the broader AF population in terms of comorbidities, disease severity, risk of MACEs, and treatment characteristics. Therefore, although higher AF burden is consistently associated with increased risk of adverse outcomes, whether reducing AF burden directly improves hard clinical endpoints remains to be established in prospective RCTs.

## 5. Imaging and Biomarkers of AF Burden

### 5.1. Imaging Markers of AF Burden

Although quantifying AF burden helps to understand the temporal extent of arrhythmia, it does not fully encompass the underlying atrial substrate that drives its development and progression. Imaging assessment may provide complementary information by identifying patients with structural and functional abnormalities associated with higher AF burden, greater arrhythmia progression, or differential response to rhythm-control therapies.

Echocardiographic assessment of LA size is routinely performed to evaluate LA remodeling and estimate AF risk [106]. Measurements that include the anteroposterior diameter and LA volume index (LAVI) reflect the underlying atrial substrate and help us identify patients with more advanced atrial disease who are more likely to experience higher rates of AF recurrence after catheter ablation, as well as increased risks of stroke and mortality [107,108,109]. However, these conventional measures may not detect early functional abnormalities. To overcome these limitations, emerging approaches based on three-dimensional LA reconstruction provide a more comprehensive assessment of atrial remodeling by capturing complex chamber geometry and regional structural characteristics [110]. Recent advances in two-dimensional speckle-tracking echocardiography (2D-STE), particularly LA strain analysis, enable more sensitive assessment of LA function. Among its three components—reservoir, conduit, and contractile strain—LA reservoir strain has emerged as the most robust marker of early LA dysfunction. Note that it often becomes impaired before significant LA enlargement occurs [111]. In the MESA (Multi-Ethnic Study of Atherosclerosis) study, reduced LA strain was independently associated with subclinical AF and increased atrial ectopy detected by prolonged monitoring. These findings support its role as a marker of an adverse atrial substrate associated with higher a AF burden [112].

LGE-CMR has emerged as an increasingly valuable imaging modality that provides a noninvasive assessment and quantification of atrial fibrosis, which is closely associated with AF development, progression, and recurrence [45,113]. Although LGE-CMR allows noninvasive detection of focal atrial fibrotic areas, its accuracy may be affected by the thin atrial wall and imaging-related motion artifacts. More recent T1 mapping approaches, including native and post-contrast techniques, enable quantitative evaluation of diffuse fibrosis with greater reproducibility [41].

In a patient cohort receiving continuous rhythm monitoring, increased LA fibrosis quantitated using LGE-CMR showed independent association with incident AF as well as higher AF burden, defined both by more frequent AF episodes and lengthier duration during each episode. These results demonstrate that the extent of atrial fibrosis, assessed by LGE-CMR, provides an important determinant of the underlying substrate which fuels AF progression [114]. Moreover, electroanatomical voltage mapping performed during ablation provides an electrophysiological evaluation of atrial remodeling, with low-voltage regions considered markers of underlying fibrotic substrate [41,115]. Combining structural information from LGE-CMR or T1 mapping with functional data from voltage mapping may improve risk stratification, facilitate personalized ablation strategies, and enhance prognostic assessment [41]. However, current variability in imaging protocols, mapping techniques, and threshold definitions limit the widespread clinical adoption of these techniques.

Epicardial adipose tissue (EAT) accumulation and phenotypic alterations are independently associated with AF, even after accounting for established clinical risk factors [116]. Computed tomography (CT) provides high spatial resolution and comprehensive cardiac coverage, and is a widely used modality for the assessment and quantification of EAT [117]. CT-derived changes in EAT attenuation correlated with histological EAT fibrosis and were associated with more advanced AF phenotypes. These findings suggest that characterization of EAT composition with the help of CT may help identify patients with an adverse atrial substrate and greater AF burden [118].

Imaging assessment of the LA appendage (LAA) plays an important role in thrombus detection and procedural planning for LAA ablation or exclusion. It can also evaluate LAA mechanical function [119,120]. Transesophageal echocardiography (TEE) remains the reference modality for thrombus detection and provides superior diagnostic accuracy compared with transthoracic echocardiography. However, its semi-invasive nature and limited availability may restrict widespread clinical use. Consequently, cardiac CT has emerged as a promising noninvasive alternative for the assessment of cardioembolic risk. Indeed, cardiac CT can detect both LAA thrombus and slow-flow, the latter representing a potential marker of impaired LAA function [121]. Evaluation of LAA morphology may provide additional prognostic information and contribute to stroke risk stratification in patients with AF [122]. Evaluation of LAA structure and function is essential for stroke risk assessment, as AF burden alone may not adequately reflect the underlying atrial substrate and thromboembolic propensity.

Overall, imaging provides complementary information on the atrial substrate beyond AF burden alone. Integration of imaging data may improve risk stratification, patient phenotyping, and the personalization of AF management.

### 5.2. Biomarkers Associated with AF Burden

Circulating biomarkers may complement AF burden assessment. They provide insight into the biological processes that contribute to increasing AF burden and progression, which include myocardial stretch, fibrosis, inflammation, renal dysfunction, oxidative stress, and myocardial injury [123,124]. Although none are currently recommended for routine assessment of AF burden, several have demonstrated significant associations with AF burden and adverse clinical outcomes.

In a multicenter cohort of patients with paroxysmal AF undergoing one year of continuous rhythm monitoring, elevated N-terminal proBNP (NT-proBNP) levels were independently associated with higher AF burden and a greater likelihood of prolonged AF episodes (≥24 h). In addition, increased concentrations of matrix metalloproteinase-2 (MMP-2), neurogenic locus notch homolog protein 3 (NOTCH3), and tumor necrosis factor receptor 2 (TNFR2) were associated with longer AF episodes, suggesting that these biomarkers reflect the biological processes underlying AF progression [125].

Similarly, in a prospective cohort of patients undergoing continuous pacemaker monitoring, higher circulating angiopoietin-2 (ANGPT2) levels were independently associated with both incident device-detected AF and increasing AF burden. Growth differentiation factor-15 (GDF-15) was not associated with AF onset but was independently related to greater AF burden, supporting a stronger role in AF maintenance than initiation. Together, these findings indicate that biomarkers of endothelial dysfunction and systemic stress may complement rhythm monitoring by identifying patients predisposed to sustained AF [126].

Conversely, the ISOLATION study demonstrated that AF burden itself is a major determinant of circulating biomarker expression. After adjustment for clinical characteristics, greater AF burden and the presence of AF at the time of blood sampling were independently associated with several biomarkers. These include bone morphogenetic protein 10 (BMP10), angiopoietin-2 (Ang-2), and fibroblast growth factor 23 (FGF23). The study also identified sex-related differences. Women exhibit higher levels of profibrotic biomarkers (BMP10 and FGF23), while men more frequently showed elevated high-sensitivity cardiac troponin T (hs-cTnT), suggesting distinct biological pathways of atrial remodeling [127].

Overall, biomarkers complement AF burden assessment by reflecting the underlying biological substrate of atrial disease. Their integration with imaging and rhythm monitoring may improve risk stratification and support more personalized management of patients with AF.

### 5.3. Integrated Imaging and Biomarker Assessment

AF burden should not be interpreted in isolation but rather integrated with imaging-derived measures of atrial remodeling and circulating biomarkers reflecting the biological substrate of atrial disease. Imaging and biomarkers provide complementary insights into structural remodeling, fibrosis, myocardial stress, inflammation, endothelial dysfunction, and atrial mechanical impairment. Consequently, patients with an identical AF burden may exhibit markedly different clinical outcomes. Therefore, combining AF burden with advanced imaging and biomarker profiling may improve patient phenotyping, facilitate identification of individuals most likely to benefit from rhythm-control interventions, enhance prediction of AF recurrence after ablation, refine thromboembolic risk assessment, and support personalized disease-modifying and anticoagulation strategies. Although prospective validation remains necessary, this multidimensional framework represents an important step toward precision medicine in AF.

## 6. AF Burden as a Therapeutic Target

### 6.1. Antiarrhythmic Drug Therapy

AAD therapy remains a cornerstone of rhythm control in AF as there is evidence that most AADs significantly reduce AF burden, irrespective of baseline AF burden, although to a lesser extent than catheter ablation [128,129].

Class Ic agents, including flecainide and propafenone, are recommended for the long-term maintenance of sinus rhythm in patients with AF and without significant structural heart disease or coronary artery disease [5]. In a large real-world cohort of more than 100,000 patients with AF, treatment with Class Ic AADs (predominantly flecainide) was associated with significantly lower all-cause mortality and fewer stroke hospitalizations than a rate-control strategy after propensity matching [130]. Although AF burden was not directly measured, the observed clinical benefits may be partly mediated by greater AF burden reduction, consistent with evidence linking lower AF burden to improved cardiovascular outcomes [131].

For patients with AF and HFrEF amiodarone, a Class III agent, is recommended to prevent AF recurrence and progression, though requiring careful consideration and regular monitoring for extracardiac toxicity [5]. A pooled analysis of the results from the AFFIRM and AF-CHF trials showed that amiodarone substantially reduced AF burden, with patients spending only 15% of follow-up time in AF, regardless of their LV function, however this burden reduction did not translate into lower hospitalization or mortality compared with rate-control therapy [132]. In the China-AF Registry, the amiodarone use was associated with higher rates of sinus rhythm maintenance compared to a non-AAD strategy, suggesting lower AF burden, but this did not translate to significant reduction in 1-year all-cause mortality [133].

Compared with amiodarone, sotalol is generally less efficacious in maintaining sinus rhythm and reducing AF burden, although data suggests similar efficacy in population with ischemic heart disease [134]. Careful patient selection and close monitoring appear to mitigate its potential safety concerns [135]. Dronedarone has similar limitations for use with sotalol. In a RCT using continuous pacemaker monitoring, dronedarone reduced AF burden by 54.4% from baseline over 12 weeks, whereas AF burden increased slightly in the placebo group. The absolute AF burden decreased by 5.5% with dronedarone compared with a 1.1% increase with placebo, demonstrating that dronedarone effectively suppresses AF burden despite being less potent than amiodarone [136]. Real world evidence supports the use of dronedarone as first line rhythm-control treatment because, compared to sotalol, it is associated with lower rates of cardiovascular hospitalization and ventricular arrhythmias [137].

Because of its selectivity, dofetilide, a class III antiarrhythmic agent, does not impair myocardial contractility, making it a suitable rhythm-control option for patients with HFrEF [138]. In 657 patients treated with dofetilide, sinus rhythm was maintained in 63% over 19 months, with AF recurrence rates comparable to amiodarone at 12 months (37% vs. 39%). These findings support that dofetilide can be considered an effective rhythm-control therapy with similar AF burden reduction, including in high-risk patients with HFrEF and other comorbidities [139].

Uncertainty remains whether beyond rhythm suppression AADs alter the natural history of AF. Early implementation of rhythm control has been implicated as an important factor underlying positive impacts on cardiovascular outcomes. However, such positive impacts seem confined predominantly to individuals presenting with early stage AF, where significant atrial remodeling and subsequent, irreversible structural changes have yet to develop [140].

Therefore, these outcomes may reflect the advantages of timely rhythm restoration and comprehensive disease management rather than a direct disease-modifying effect of AADs alone. Accordingly, contemporary assessment of AAD efficacy should extend beyond binary recurrence endpoints to include quantitative reductions in AF burden, symptom improvement, and QoL outcomes.

### 6.2. Catheter Ablation and AF Burden

Catheter ablation is one of the most effective rhythm-control strategies for achieving sustained reductions in AF burden [141]. Indeed, it has demonstrated superiority over AAD therapy in maintaining rhythm control and improving long-term outcomes [142].

In the CIRCA-DOSE trial, catheter ablation achieved more than a 98% reduction in AF burden relative to baseline despite the occurrence of recurrent arrhythmia in some patients, showing that recurrence alone does not adequately reflect treatment success [143]. The CABANA trial reinforced these findings and showed that catheter ablation resulted in a sustained and significantly lower AF burden than drug therapy throughout 5 years of follow-up (6.3% vs. 14.4% at 12 months), irrespective of baseline AF pattern [144]. Another RCT comparing catheter ablation with AAD therapy found that ablation resulted in a significantly lower AF burden at 12 months and greater improvements in QoL, further supporting the clinical relevance of reducing AF burden rather than focusing solely on arrhythmia recurrence [145].

Clinical relevance of AF burden reduction following catheter ablation is significant as lower AF burden has been associated with improvements in symptom severity, QoL, functional capacity, and daily physical activity. Additionally, decreases in AF burden following ablation have been demonstrated to be accompanied by decreases in overall health care utilization, including hospitalizations and emergency department or outpatient clinic visits. This further underscores AF burden as an important therapeutic goal for patients undergoing AF ablation [141].

Despite these findings, important methodological considerations remain. Clinically meaningful thresholds for AF burden reduction after catheter ablation have not been universally established. Studies continue to use heterogeneous monitoring strategies and endpoint definitions. Therefore, although AF burden provides a more comprehensive assessment of procedural efficacy than binary recurrence alone, its role as a standardized endpoint in ablation trials requires further validation.

AF burden reduction benefits extend beyond symptom control as emerging data explore its relationship with thromboembolic risk. Although CABANA did not demonstrate a reduction in stroke risk with ablation, EAST-AFNET 4 suggested that early rhythm-control strategies, potentially through earlier suppression of AF burden, may reduce stroke incidence [140,144]. Catheter ablation is also associated with a reduced risk of stroke as shown in a recent meta-analysis incorporating RCTs [146]. These findings have renewed interest in the relationship between post-ablation AF burden, thromboembolic risk, and the role of long-term oral anticoagulation after apparently successful rhythm control.

In the OCEAN trial, patients without AF recurrence for at least one year after catheter ablation were randomized to rivaroxaban or aspirin. Thromboembolic event rates were low and did not differ significantly between the two groups, whereas bleeding events occurred more frequently with rivaroxaban. Although the trial was underpowered, these findings suggest that patients with sustained suppression of AF, and presumably very low AF burden, may derive limited additional benefit from continued anticoagulation [147]. The ALONE-AF trial that randomized arrhythmia-free patients one year after AF ablation to continue or discontinue oral anticoagulation also showed that thromboembolic events were rare in both groups, while bleeding was more frequent with continued anticoagulation. These studies are based on the absence of AF recurrence rather than AF burden and should not be interpreted as evidence that AF burden alone can guide our anticoagulation decisions. However, the findings support further investigation of AF burden as a complementary factor in individualized anticoagulation strategies [148]. It is important to emphasize that these findings cannot be generalized to higher-risk patients and ongoing rhythm surveillance remains essential after anticoagulation withdrawal.

The RAFT-AF trial evaluated an ablation-based rhythm-control strategy in individuals with high-burden AF and HF. Ablation resulted in improvements in LV function, NT-proBNP levels, exercise capacity, and QoL compared with rate control, although the primary endpoint of mortality or HF events did not reach statistical significance [149]. These findings are in keeping with the suggestion that reducing AF burden may lead to favorable cardiac remodeling, especially in patients with functional impairment. Finally, recent real-world data demonstrated that patients with persistent AF undergoing catheter ablation had similar risks of mortality and hospitalization compared with those with paroxysmal AF after adjustment for comorbidities, suggesting that long-term outcomes may be driven more by underlying disease burden than AF pattern alone. These findings reinforce the importance of AF burden as a therapeutic target rather than relying solely on conventional AF classification [150].

Importantly, catheter ablation has the potential not only to reduce AF burden but also to reverse atrial remodeling in selected patients [151,152]. As electroanatomical mapping and CMR imaging suggest, successful rhythm stabilization after ablation has the potential to partially reverse electrical and structural atrial alterations [153]. These findings support the concept that ablation may have disease-modifying effects in AF by attenuating atrial remodeling. Although radiofrequency and cryoballoon ablation remain well-established thermal modalities, pulsed field ablation (PFA) has emerged as a non-thermal alternative that induces preferential myocardial cell death and minimizes collateral tissue injury [154,155]. Whether these mechanistic differences translate into greater long-term substrate modification or AF burden reduction remains an area of active investigation. A recent meta-analysis of 32 RCTs that included 7226 patients found comparable efficacy across ablation modalities but reported greater AF burden reduction with PFA compared with radiofrequency and cryoballoon ablation. This suggests a potential role for PFA in reducing residual AF burden [156].

Furthermore, there is an increasing need for future studies to clarify the optimal timing of catheter ablation to reduce AF progression and lifetime burden while ensuring equitable access to AF therapies across diverse patient populations. Supporting this concept, a recent meta-analysis of 28 studies including 41,431 patients found that ablation performed within 1 year of AF diagnosis was associated with a lower risk of AF recurrence, repeat ablation, cardioversion, and cardiovascular hospitalization compared with delayed ablation, suggesting that earlier rhythm control may limit AF burden and disease progression [157].

### 6.3. Lifestyle Strategies to Reduce AF Burden

Multiple modifiable lifestyle and cardiovascular risk factors, including DM, dyslipidemia, obesity, hypertension, obstructive sleep apnea, excessive alcohol consumption, physical inactivity, and smoking can influence the onset and progression of AF. Targeted management of these factors has been associated with reduced AF symptoms, improved rhythm control, and potential reversal of atrial cardiomyopathy [158].

Lifestyle interventions have emerged as potential disease-modifying strategies, particularly alongside catheter ablation, and are the focus of ongoing research. Current studies are evaluating the optimal timing of lifestyle optimization relative to ablation and whether lifestyle changes alone or combined with AADs can provide meaningful rhythm-control benefits. The PRAGUE-25 trial found that catheter ablation was more effective than lifestyle intervention plus AAD therapy for maintaining sinus rhythm, whereas the SORT-AF trial did not demonstrate a significant reduction in AF burden among patients who achieved weight loss after ablation [159,160]. Importantly, recent evidence suggests that comprehensive lifestyle modification, including home sleep apnea assessment, weight loss, moderation of alcohol consumption, smoking cessation, and appropriate management of hypertension and dyslipidemia before ablation may reduce the need for cardioversion and repeat ablation procedures by approximately 50% during the first year after treatment [161]. These conflicting evidences emphasizes the need for further studies to define the role and especially the timing of lifestyle interventions that lead to optimal AF management.

### 6.4. Cardiometabolic and Neurohormonal Therapies as Emerging AF Burden Modulators

Cardiometabolic therapies, particularly sodium-glucose cotransporter-2 inhibitors (SGLT2is) and glucagon-like peptide-1 receptor agonists (GLP-1RAs) have received increased attention for their potential role of reducing AF burden and modifying disease progression. Although originally developed for the treatment of DM, these agents have demonstrated cardiovascular benefits that extend beyond glycemic control, prompting investigation into their potential role in AF prevention and burden reduction [162].

The beneficial effects of SGLT2 inhibitors on AF may result from a combination of direct cellular effects and indirect systemic mechanisms. These include modulation of intracellular Ca^2+^ handling through regulation of Ca/calmodulin-dependent protein kinase II (CaMKII) activity, reduction of oxidative stress, improvement of myocardial metabolism, attenuation of inflammation and adverse cardiac remodeling, suppression of sympathetic activity, and favorable effects on body weight, blood pressure and overall organ function [163]. As shown in a meta-analysis of 31 RCTs, SGLT2is were associated with a lower risk of AF events, suggesting a potential role in reducing AF burden, although studies with AF burden as a primary endpoint remain limited [164]. Furthermore, a recent meta-analysis showed that SGLT2i therapy was associated with reduced AF recurrence after ablation, particularly during the first 12–18 months of follow-up, supporting their use as a potential adjunctive strategy to improve rhythm control [165]. However, despite these promising findings, further dedicated RCTs are required to confirm AF-specific treatment recommendations.

Regarding GLP-1 RAs, emerging evidence indicates that these agents may exert favorable effects on AF risk and potentially contribute to AF burden reduction. A recent meta-analysis of 24 RCTs found that GLP-1RAs and co-agonists were associated with an 18% lower risk of incident AF among individuals with overweight or obesity, suggesting a potential role in reducing AF burden through weight loss, improved glycemic control, reductions in EAT, attenuation of systemic inflammation, modulation of autonomic tone, amelioration of diastolic dysfunction and LA pressure, suppression of LA fibrosis, and inhibition of myofibroblast differentiation [166]. Among those patients receiving AF ablation, another recent meta-analysis indicated that the use of GLP-1 RA was associated with reduced AF recurrence, suggesting a potential role in enhancing rhythm control and lowering AF burden. The results demonstrate the potential of using cardiometabolic interventions as an emerging treatment option for mitigating AF burden. However, prospective RCTs are needed to validate these findings [167].

Pharmacological targeting of the renin–angiotensin–aldosterone system (RAAS) has shown a modest ability to reduce the development of new-onset AF, with the greatest benefits observed in patients with concomitant HF. Evidence from RCT meta-analyses indicates that angiotensin-converting enzyme inhibitors (ACEi) and angiotensin receptor blockers (ARBs) are associated with a lower incidence of AF, although their effects are less evident in patients without significant structural cardiac disease [168]. Furthermore, in hypertensive patients, ACEi and ARBs have showed a reduction in AF recurrence compared with other antihypertensive therapies. These findings suggest that RAAS inhibition may help limit AF burden progression [169].

Mineralocorticoid receptor antagonists (MRAs) may provide greater antiarrhythmic effects, particularly in reducing AF recurrence and among individuals with impaired LV function [170,171,172,173]. These therapies may attenuate atrial remodeling by limiting LA enlargement and fibrotic pathways, as demonstrated in experimental and clinical studies [174]. However, their impact appears limited in paroxysmal AF, where structural remodeling and RAAS activation are typically less pronounced [41].

### 6.5. Interventional Strategies for AF Burden Reduction Beyond Ablation

Surgical and hybrid strategies represent important alternative options for selected patients with advanced or persistent AF, particularly when the goal is sustained reduction of AF burden. The Cox-Maze IV (CMP-IV) procedure remains the surgical gold standard for patients with AF undergoing cardiac surgery, providing durable rhythm control through modifying the arrhythmogenic substrate [175,176]. Freedom from atrial tachyarrhythmias remained 92%, 84%, and 77% at 1, 5, and 10 years, respectively, as showed in a large long-term cohort, however the population was largely comprised patients with non-paroxysmal AF and prior failed catheter ablation. These findings, although influenced by parameters such as AF type, LA enlargement, and age, indicate that extensive surgical substrate modification can achieve sustained suppression of AF [177].

Hybrid convergent procedures, which combine minimally invasive epicardial ablation with endocardial catheter ablation, have also demonstrated encouraging results in patients with persistent and long-standing persistent AF [178,179]. Long-term follow-up showed that more than half of patients maintained an AF burden < 1% on the 7-day Holter ECG approximately 8 years after the procedure, while two-thirds remained in sinus rhythm. Although repeat catheter ablation was required in a proportion of patients to preserve rhythm control, these data suggest that hybrid approaches can achieve meaningful and durable reductions in AF burden in patients with more advanced atrial disease [180].

As previously discussed, AF and mitral regurgitation (MR) frequently coexist, with each condition capable of contributing to the development and progression of the other [181,182]. Mitral transcatheter edge-to-edge repair (M-TEER) is now considered a safe, effective, and minimally invasive treatment for patients with severe MR who are at high surgical risk, especially benefiting the cohorts of patients with secondary MR associated with HF [183]. Emerging evidence from a multicenter cohort of patients with severe MR undergoing M-TEER suggested that AF burden was significantly reduced at 3 months after the procedure, although this benefit was not sustained at 12 months. This highlights that although M-TEER may favorably influence AF burden there is a need for larger prospective studies to clarify the long-term effects of MR correction on AF burden and related clinical outcomes [184].

Atrial remodeling driven by chronic pressure and volume overload may be at least partially reversible when the underlying structural abnormalities are effectively treated. In patients with HFrEF, CRT has been associated with reductions in LA volume and improvements in atrial reservoir function within months of implantation, likely reflecting reverse LV remodeling and improved cardiac loading conditions [185]. However, despite these favorable structural and functional effects, CRT has not consistently translated into reductions in AF burden. In one study, CRT improved cardiac function and promoted reverse ventricular remodeling but did not significantly reduce AF burden compared with controls, although it appeared to delay the onset of new AF in patients without a prior history of AF [186].

Collectively, surgical and hybrid strategies further support AF burden, rather than arrhythmia recurrence alone, as a clinically relevant therapeutic target (Table 1).

### 6.6. Proposed Integrated Anticoagulation Framework

Although current guidelines recommend anticoagulation primarily according to the CHA_2_DS_2_-VASc score, growing evidence suggests that future treatment decisions may benefit from incorporating AF burden into a broader, multidimensional assessment of thromboembolic risk [5,68]. AF burden should be viewed as one component of an integrated risk model that reflects both the arrhythmic substrate and the patient’s overall cardiovascular profile and not as an isolated determinant.

It is important to note that anticoagulation decisions should distinguish subclinical AHRE detected by implanted devices from clinically diagnosed AF because these entities may represent different thromboembolic risk profiles. Recent trials have provided important but nuanced insights in this field. In ARTESIA, apixaban reduced ischemic stroke or systemic embolism in patients with device-detected subclinical AF. However, this benefit was accompanied by an increased risk of major bleeding [66]. Conversely, NOAH-AFNET 6 did not demonstrate a net clinical benefit of anticoagulation in patients with AHRE without established AF [65]. Therefore, the decision to initiate anticoagulation in patients with AHRE should remain individualized, and we have to consider episode characteristics, baseline stroke risk, and bleeding risk. Likewise, decisions regarding continuation or discontinuation of oral anticoagulation after apparently successful AF ablation should remain individualized. As discussed above, recent RCTs (OCEAN and ALONE-AF) suggest that selected patients with durable rhythm control may derive limited benefit from continued anticoagulation; however, current evidence remains insufficient to support AF burden alone as determinant of anticoagulation therapy [147,148].

In this framework, anticoagulation decisions are guided by four complementary domains: (1) clinical stroke risk, assessed using validated scores such as CHA_2_DS_2_-VASc; (2) AF burden, quantified by continuous monitoring in terms of episode duration, frequency, and cumulative burden; (3) atrial substrate, including structural and functional markers of atrial cardiomyopathy such as LA enlargement, atrial fibrosis, reduced atrial strain, and impaired LA appendage function; and (4) circulating biomarkers, including NT-proBNP, hs-cTnT, GDF-15, and inflammatory markers, which may identify patients with ongoing myocardial injury or advanced atrial disease.

Through this integrated approach the thromboembolic risk is calculated from the association between patient-related factors and the severity of the atrial disease process rather than AF burden alone. This multidimensional model may enable more individualized anticoagulation strategies as the continuous rhythm monitoring, advanced cardiac imaging, and biomarker-guided risk assessment become increasingly available. This approach may optimize stroke prevention and minimize unnecessary bleeding risk, particularly in clinical scenarios where the benefit of long-term anticoagulation remains uncertain. Although prospective validation is still required, this framework represents a logical step toward precision medicine in AF.

Taken together, the available evidence supports an integrated, patient-centered approach to AF burden reduction with the goal of improving long-term clinical outcomes. Such a multidimensional framework acknowledges that AF burden is one component of a broader atrial disease process and may ultimately facilitate more personalized anticoagulation strategies while balancing stroke prevention against bleeding risk. Although further prospective studies are warranted, combining early rhythm-control interventions, comprehensive risk-factor management, evidence-based cardiometabolic therapy, and individualized thromboembolic risk assessment represents a promising framework for disease modification and precision medicine in AF.

## 7. AF Burden as a Clinical Trial Endpoint

### 7.1. Limitations of the 30-s Recurrence Definition

The definition of AF recurrence as any episode lasting ≥ 30 s was first described in early catheter ablation studies. This duration was chosen primarily for methodological consistency and because episodes of this duration could be reliably detected and distinguished from artifact on electrocardiographic monitoring. It was later broadly adopted by consensus statements to provide a standardized endpoint for clinical trials as it facilitated comparisons across studies and regulatory evaluation of emerging rhythm-control therapies [7,187,188].

The 30-s definition has important limitations as it classifies all qualifying episodes equally, regardless of their duration, frequency, or clinical significance [131]. With this definition a single asymptomatic 30-s episode is considered equivalent to frequent or sustained AF and would lead to classify treatment as a failure despite minimal AF burden.

Moreover, interventions that substantially reduce AF burden—for example, from persistent or daily episodes to only occasional brief recurrences—may be considered unsuccessful even though they a marked reduction in arrhythmia is achieved. As a result, this endpoint underestimates clinically meaningful improvements and limits assessment of therapeutic efficacy [131,188].

Finally, the 30-s recurrence definition correlates poorly with patient-centered outcomes [4,5]. AF symptoms, QoL, functional capacity, and healthcare utilization are influenced more by the overall burden and pattern of AF than by the mere occurrence of a brief episode. Similarly, emerging evidence suggests that adverse outcomes, including HF and thromboembolic risk, are more closely associated with cumulative AF burden than with isolated short-lasting recurrences. These limitations have prompted increasing interest in AF burden as a more clinically relevant measure of treatment success, particularly in the era of continuous rhythm monitoring [131,188,189].

### 7.2. Advantages of Burden-Based Endpoints

AF burden metrics have been shown to be more sensitive indicators of clinically relevant reductions in AF compared with dichotomous recurrence rates, offering important guidance for future management approaches within current clinical practice [190].

In addition, incorporation of an AF burden approach may enhance trial efficiency through reduction of variability and enhancement of statistical power, which could lead to decreased numbers needed per study arm to establish proof-of-concept therapy effectiveness. Importantly AF burden may better reflect patient-centered outcomes, as reductions in arrhythmia exposure are more closely associated with improvements in symptoms, QoL, healthcare utilization, and potentially AF-related cardiovascular outcomes than isolated recurrence events.

### 7.3. Standardization Needs

Broader implementation of AF burden as a trial endpoint requires harmonized definitions and reporting standards. It is challenging to assess AF burden when patients develop sustained symptomatic AF requiring interventions such as electrical cardioversion or repeat catheter ablation, as these procedures deliberately terminate AF. Therefore, the subsequent AF burden may reflect the timing of physician- or patient-driven intervention rather than the natural treatment effect. Clinical trialists should prespecify how these patients will be handled in the primary analysis—for example, by censoring AF burden measurements after intervention, assigning a predefined nominal burden, or incorporating the intervention into a composite endpoint. Such a transparent and consistent handling of these cases is essential to minimize bias and ensure comparability across studies [3].

Clinical trials should also prespecify the type of monitoring device, duration of follow-up, and minimum analyzable recording time, as these factors substantially influence burden estimates.

Standardized reporting should include absolute AF burden, relative change from baseline, episode characteristics, and clinically relevant thresholds for treatment response. Importantly, currently proposed AF burden thresholds are largely derived from individual studies and should be interpreted as study-specific estimates rather than universally applicable biological cut-offs, as current evidence suggests a continuous relationship between AF burden and clinical outcomes. The management of rhythm-control interventions occurring during follow-up, including cardioversion, repeat ablation, and initiation or escalation of AAD therapy, should be clearly defined, as these events can substantially influence measured AF burden [3].

A unified framework for AF burden assessment will facilitate comparison across clinical trials, improve interpretation of therapeutic effects, and support the integration of AF burden as a potentially clinically relevant endpoint in future rhythm-control studies pending further validation.

## 8. Future Directions

The increasing availability of continuous rhythm monitoring along with the advances in digital health are expected to change AF management from a binary classification of recurrence toward a more precise rhythm-control strategy based on AF burden [3,4]. Integration of wearable technologies, implantable monitoring devices, and remote patient management platforms may enable continuous, personalized assessment of AF burden and facilitate earlier therapeutic intervention [43]. Instead of focusing only on the presence or absence of recurrent AF, future care may include burden trajectories to guide treatment escalation, including optimization of AADs, repeat catheter ablation, and individualized follow-up intensity.

**Figure 2:**
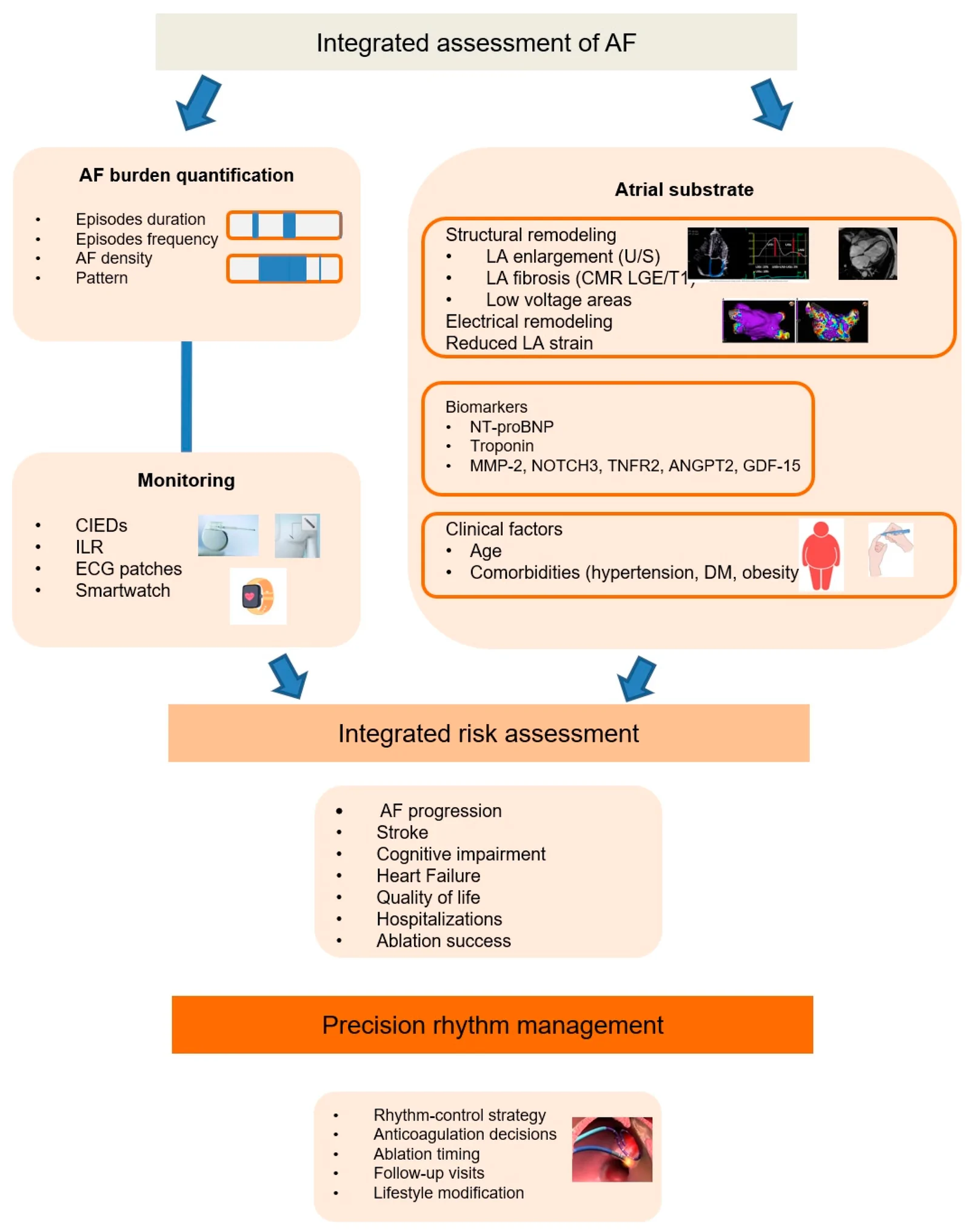
Integrated assessment of AF burden and atrial disease. AF burden reflects the quantitative expression of arrhythmia (episode frequency, duration, density, and percentage of time in AF), whereas atrial disease represents the underlying structural, electrical, and functional atrial substrate. Combining continuous rhythm monitoring with imaging, electroanatomical mapping, biomarkers, and clinical characteristics provides a comprehensive assessment of disease severity, enabling improved prediction of stroke, HF, AF progression, symptoms, and catheter ablation outcomes, while supporting individualized rhythm-control strategies. AF, atrial fibrillation; LA, left atrial; U/S, ultrasound; CMR, cardiovascular magnetic resonance; LGE, late gadolinium enhancement; CIEDs, cardiac implantable electronic devices; ILR, implantable loop recorder; ECG, electrocardiogram; NT-proBNP, N-terminal pro–B-type natriuretic peptide; MMP-2, matrix metalloproteinase-2; NOTCH3, neurogenic locus notch homolog protein 3; TNFR2, tumor necrosis factor receptor 2; ANGPT2, angiopoietin-2; GDF-15, Growth differentiation factor-15; DM, diabetes mellitus.

The clinical use of AF burden by identifying complex temporal patterns that are not apparent using conventional analyses is expected to be revolutionized by the advances in AI and advanced data analytics. Machine learning models integrating AF burden with clinical characteristics, imaging, biomarkers, and genetic data may improve prediction of AF progression, stroke risk, HF deterioration, cognitive decline, and long-term ablation outcomes, thereby supporting individualized risk stratification and treatment decisions [191] (Figure 2).

Despite the ongoing substantial progress, there are several important knowledge gaps that will need to be investigated. Thresholds for AF burden reduction that translate into improved patient outcomes have not been clearly established and similarly, the AF burden at which anticoagulation should be initiated or discontinued remains uncertain, an issue particularly relevant in patients with device-detected or low-burden AF. Additional burden metrics such as AF density and temporal distribution of episodes, need to be further validated to determine whether they provide incremental prognostic information beyond overall burden alone. AF burden is also a dynamic parameter that evolves over time in response to aging, comorbidities, and therapeutic interventions, emphasizing the need for longitudinal rather than single time-point assessment. Future burden-guided clinical trials will be essential to determine whether individualized strategies based on AF burden trajectories improve patient outcomes.

The adoption of standardized AF burden definitions and reporting methodologies will facilitate comparisons across studies, enable identification of actionable burden thresholds, and accelerate the integration of AF burden into routine clinical practice and future guideline recommendations, while prospective studies are needed to establish whether AF burden can serve as an independent therapeutic target beyond its role as a marker of disease severity and treatment response.

## 9. Conclusions

AF burden has emerged as a more comprehensive and clinically meaningful metric of AF compared to the conventional classification based on AF pattern or binary recurrence. The association of higher AF burden with poor clinical outcomes including an increased risk of stroke, HF, hospitalization, disease progression, and impaired QoL, highlights its value as a marker of disease severity and treatment response and indicates the importance of rhythm-control therapies that can achieve substantial reductions in AF burden even if isolated recurrence is found. These findings underscore the rationale for abandoning dependence on the conventional AF recurrence endpoint (>30 s) as the principal determinant of therapeutic success. Incorporation of AF burden into clinical trials provides a more sensitive assessment of therapeutic efficacy that can better capture outcomes that matter to patients and may support individualized treatment plans based on rhythm-control strategies and longitudinal risk assessment. Future efforts should focus on standardizing definitions, leveraging existing capabilities for monitoring of AF burden, identifying clinically actionable thresholds of AF burden and conducting prospective burden-guided RCTs to determine whether AF burden can serve as an independent therapeutic target and define its role in both AF research and patient management.

## Figures and Tables

**Table 1:** Effects of rhythm-control and adjunctive therapies on AF burden and clinical outcomes.

Therapeutic Strategy	Representative Evidence	Effect on AF Burden	Clinical Implications	References
Class Ic AADs (flecainide, propafenone)	Retrospective observational cohort studies	Reduction in AF burden	For long-term maintenance of sinus rhythm in AF patients without significant structural heart disease or CAD; associated with lower mortality and stroke hospitalization compared with rate control.	[125,126,127]
Amiodarone	Post hoc pooled analysis of RCTs (AFFIRM, AF-CHF), retrospective observational cohort study	Reduction in AF burden	Effective AAD, particularly in HFrEF, although burden reduction did not translate into lower mortality or hospitalization.	[129,130]
Dronedarone	Pacemaker-monitoring RCT, meta-analysis of retrospective observational databases	~54% relative reduction in AF burden	Effectively reduces AF burden, though less potently than amiodarone. Lower cardiovascular hospitalization and ventricular arrhythmia rates than sotalol	[133,134]
Sotalol	RCT	Moderate to substantial AF burden reduction	Sotalol effectively maintains sinus rhythm in AF, with comparable efficacy to amiodarone in ischemic heart disease, improving QoL and exercise capacity.	[137]
Dofetilide	Retrospective observational cohort study	Reduction in AF burden	Dofetilide maintains sinus rhythm effectively without impairing contractility, with AF recurrence rates comparable to amiodarone, including in HFrEF and high-risk patients.	[135,136]
Catheter ablation	RCTs, meta-analysis	Significant reduction of AF burden; consistently lower burden than AADs	Improves symptoms, QoL, exercise capacity, LVremodeling, reduces healthcare utilization and risk of stroke. Selected low-burden AF patients with successful ablation may discontinue anticoagulation with continued rhythm monitoring.	[138,139,140,141,142,143,144,145,146]
Lifestyle and risk-factor modification	RCTs	Variable reduction; greater benefit when comprehensive and initiated before ablation	May reduce repeat ablation and cardioversion, supporting disease modification despite heterogeneous trial results.	[151,152,153]
Cardiometabolic therapies (SGLT2 inhibitors, GLP-1 receptor agonists)	Meta-analyses	Lower AF recurrence, suggesting reduced AF burden	Promising adjunctive therapies through metabolic and structural remodeling; AF burden-specific RCTs remain limited.	[157,158,159,160]
RAAS inhibition (ACEi/ARB, MRAs)	Meta-analyses	Reduced AF recurrence and slower AF progression, particularly in HF	May attenuate atrial remodeling and complement rhythm-control strategies.	[161,162,163,164,165]
Cox-Maze IV,	Retrospective comparative cohort studies, prospective observational cohort study, retrospective observational cohort study	Sustained reduction of AF burden with long-term arrhythmia-free survival	Durable rhythm-control option for selected AF patients, especially those undergoing concomitant surgery or after failed catheter ablation.	[167,168,169]
Convergent ablation	RCT, meta-analysis, retrospective observational cohort study	Durable reduction in AF burden with sustained sinus rhythm in advanced AF	An efficient option for persistent/long-standing persistent AF when catheter ablation alone is insufficient.	[170,171,172]
M-TEER	Retrospective observational cohort study	Short-term AF burden reduction after M-TEER, with uncertain long-term durability	Potential adjunctive rhythm benefit of MR correction; further studies needed to define clinical role.	[176]
CRT	Prospective observational cohort study, retrospective observational matched cohort study	Improves atrial remodeling but has limited impact on established AF burden	May reduce AF development in selected patients, but additional rhythm-control strategies may be required.	[177,178]

AF, atrial fibrillation; AADs, antiarrhythmic drugs; CAD, coronary artery disease; HFrEF, heart failure with reduced ejection fraction; RCTs, randomized controlled trials; QoL, quality of life; SGLT2, sodium-glucose cotransporter 2; GLP-1, glucagon-like peptide-1; RAAS, renin–angiotensin–aldosterone system; ACEi, angiotensin-converting enzyme inhibitors; ARB, angiotensin receptor blockers; MRAs, mineralocorticoid receptor antagonists; HF, heart failure; LV, left ventricular; M-TEER, mitral transcatheter edge-to-edge repair; CRT, cardiac resynchronization therapy.

## Data Availability

All data generated in this research is included within the article.
